# Supplementary material for: Dysregulated Host Responses Underlie 2009 Pandemic Influenza-Methicillin Resistant Staphylococcus aureus Coinfection Pathogenesis at the Alveolar-Capillary Barrier
Source: Cells. 2020 Nov 13;9(11):2472. doi: 10.3390/cells9112472 (PMC7696554; doi:10.3390/cells9112472)
Supplement: Supplementary file 1 [file cells-09-02472-s001.pdf]

Figure S1: Alveolar-capillary barrier co-culture model schematic. Figure generated with BioRender. The basal side of 0.4 µm transwell inserts were coated with GelTrex LDEV-Free Reduced Growth Factor Basement Membrane Matrix and rested basal side up for 1 h at 37°C and 5% CO2. Transwell inserts were turned apical side up, the apical side coated with GelTrex, and rested for 1 h at 37°C and 5% CO2. Transwell inserts were turned basal side up, the basal side of the transwell inserts seeded with HPMEC at a concentration of 1.5 × 10^5 cells/mL (4.5x10^4 cells/cm2) in a 1:1 mix of HPAEpiC and HPMEC media, and rested for 3 h at 37°C and 5% CO2. Transwell inserts were turned apical side up and HPAEpiC were seeded on the apical side at a concentration of 3 x 10^5 cells/mL (9x 10^4 cells/cm2) in a 1:1 mix of HPAEpiC and HPMEC media. After 24 h, media was removed from the upper compartment of the transwell insert, to allow primary epithelial cells to grow at the air-liquid interface. Media in the lower compartment was refreshed with a 1:1 mix of HPAEpiC and HPMEC media. Cells were permitted to grow to confluency for 14 days, with media in the lower compartment being refreshed every second day.

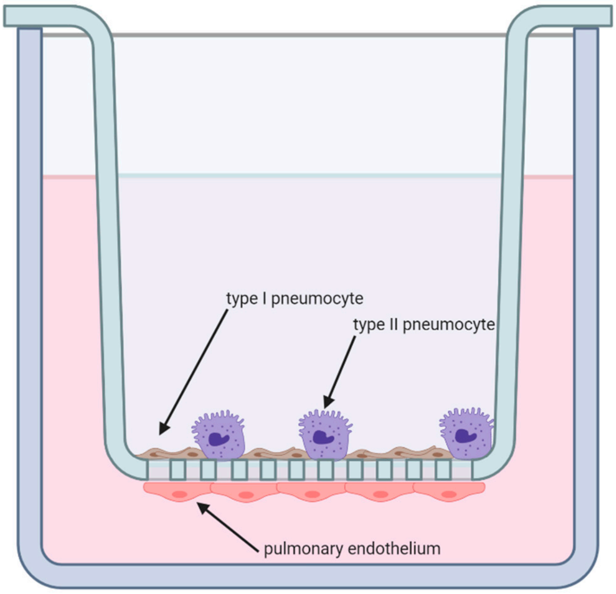

Figure S2: Viral loads decrease during MRSA co-infection in the alveolar-capillary barrier model. Supernatants were collected by addition of PBS to the apical surface of the alveolar epithelial cells and harvested for isolation of viral RNA prior to quantification by RT-qPCR. Error bars represent SEM calculated from at least three biological replicates.

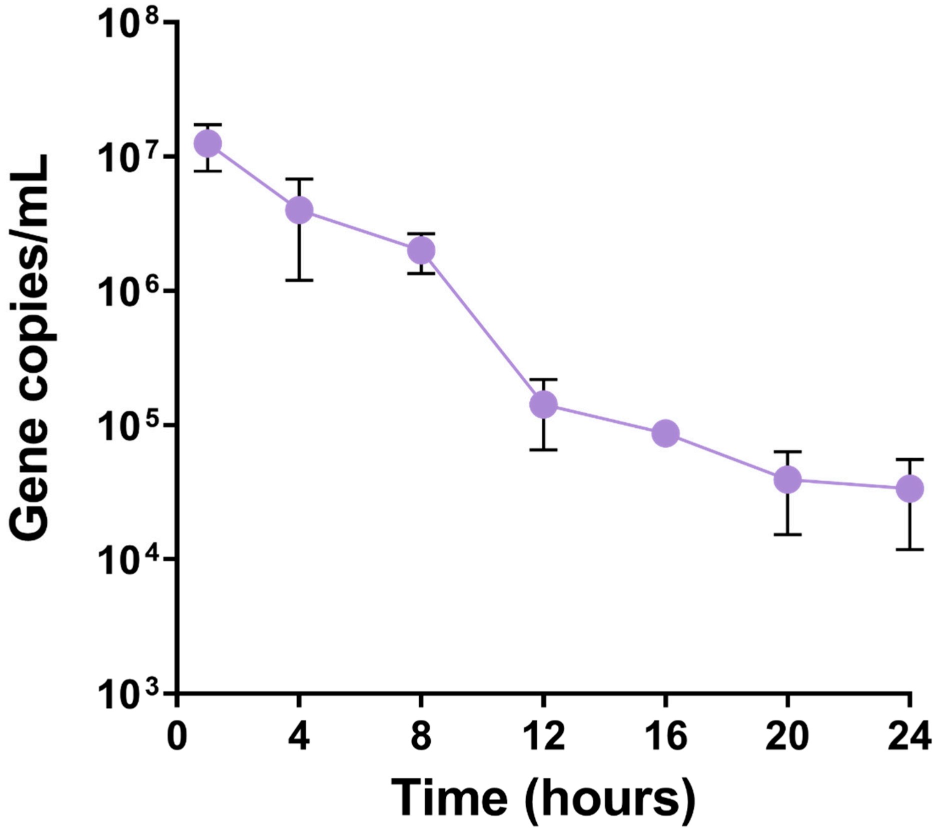

Table S1: MRSA Virulence Factor Primer Sequences. Primers were designed and selected using PrimerQuest.

Gene	Function	Primer Sequences (5′ to 3′)
Coa	protects bacteria from phagocytosis by forming fibrin clots	F – TTCCACAGGGCACAATTACA<br>R-TCGGGACCTTGAACGATTTC
EbpS	binds elastin to facilitate colonization of injured tissues	F – GGTGAACCTGAACCGTAGTATT<br>R - CAGCAACAACAACGTCAAGG
Eno	forms plasmin to digest fibrin clots which keep the bacterium localized	F - TGGTTACAAACCAGGTGAAGAA<br>R - CGCCTTCGAACTTACTGTAGTC
FnbA	binds extracellular matrix components to mediate endothelial cell adhesion	F - CCCATTTCCGTTCGCTTTATTAC<br>R - GTAGGACATCCAGAGCAACTTTA
FnbB	binds fibronectin and elastin to mediate adhesion to endothelial cells	F - TGTCGCGCTGTATGATTGT<br>R - GTAGAGGAAAGTGGGAGTTCAG
Hla	forms pores in the cytoplasmic membrane resulting in lysis	F - CTGTAGCGAAGTCTGGTGAAA<br>R - AGATTCTTGGAACCCGGTATATG
HlgA	forms pores in the cytoplasmic membrane resulting in lysis	F - CCAGCAGCACGAGACTATTT<br>R - CACCTTTACCTCTTTCGTGTGA
IcaA	mediates intercellular adhesion and biofilm formation	F - GCAGTAGTTCTTGTCGCATTTC<br>R - GTTGGGTATTCCCTCTGTCTG
IcaB	mediates intercellular adhesion and biofilm formation	F - AGCCTATCCTTATGGCTTGATG<br>R - GAGTTCGGAGTGACTGCTTT
Sbi	mediates Fab-independent formation of IgG insoluble complexes	F - AGCCAACAAGTTTGGGTAGAA<br>R - CGTGTGGTGCTTTGTTATCTTG
Sek	enterotoxin with superantigen activity	F – ATCGACATCCAAATGGAATTTCTC<br>R - CTACACAGGAGATGATGGGTTAC
Seq	enterotoxin with superantigen activity	F -GTAGAAACCTCGTCTGTAGATATAGTG<br>R - GGAATTACGTTGGCGAATCAAA
Spa	forms IgG insoluble complexes to impede phagocytosis; binds airway epithelial cells	F – GCTGCACCTAAGGCTAATGATA<br>R - GATAAGAAGCAACCAGCAAACC

Table S2: Upregulated signaling pathways in pH1N1-MRSA co-infected alveolar epithelial cells at the alveolar-capillary barrier as determined by temporal kinome analysis. Pathways represent those that were found to be activated in pH1N1-MRSA co-infected cells as compared to mock-infected controls at 24 h post-MRSA addition. Thresholds for pathways over-representation analysis were fold-change ≥1.5, p-value ≤ 0.05.

Pathway Name	Pathway uploaded proteins	Genes in InnateDB for this entity	Pathway up-regulated genes count	Pathway up-regulated p-value	Gene Symbols
Estrogen responsive protein efp controls cell cycle and breast tumors growth	4	4	3	1.78E-04	CDK1; CDK2; CDK6; CDK7;
Cyclin A/B1 associated events during G2/M transition	5	5	3	4.35E-04	CDC25A; CDK1; CDK2; CDK7; PPP2CA;
G1/S Transition	6	6	3	8.50E-04	CCNE1; CDC25A; CDK1; CDK2; CDK7; PPP2CA;
Cyclins and cell cycle regulation	7	7	3	0.001452	CCND1; CCNE1; CDC25A; CDK1; CDK2; CDK6; CDK7;
Mitotic G1-G1/S phases	8	8	3	0.002268	CCND1; CCNE1; CDC25A; CDK1; CDK2; CDK6; CDK7; PPP2CA;
G2/M Checkpoints	3	3	2	0.003969	CDC25A; CDK1; CDK2;
G2/M Transition	10	10	3	0.004632	CDC25A; CDK1; CDK2; CDK7; CSNK1D; CSNK1E; EP300; PPP1R12A; PPP2CA; PRKACA;
Mitotic G2-G2/M phases	10	10	3	0.004632	CDC25A; CDK1; CDK2; CDK7; CSNK1D; CSNK1E;

					EP300; PPP1R12A; PPP2CA; PRKACA;
Cell Cycle Checkpoints	4	4	2	0.007759	CCNE1; CDC25A; CDK1; CDK2;
Cyclin A:Cdk2-associated events at S phase entry	4	4	2	0.007759	CCNE1; CDC25A; CDK2; CDK7;
Cyclin E associated events during G1/S transition	4	4	2	0.007759	CCNE1; CDC25A; CDK2; CDK7;
D4gdi signaling pathway	4	4	2	0.007759	CASP1; CASP3; JUN; RHOA;
ERK1 activation	4	4	2	0.007759	CDK1; JAK1; JAK2; MAP2K1;
G0 and Early G1	4	4	2	0.007759	CCNE1; CDC25A; CDK1; CDK2;
IRAK1 recruits IKK complex	4	4	2	0.007759	CHUK; PELI1; PELI2; TRAF6;
IRAK1 recruits IKK complex upon TLR7/8 or 9 stimulation	4	4	2	0.007759	CHUK; PELI1; PELI2; TRAF6;
Internal ribosome entry pathway	4	4	2	0.007759	CASP1; CASP3; CASP6; CASP8;
Regulation of cell cycle progression by plk3	4	4	2	0.007759	CASP3; CASP6; CDK1; CHEK2;
p53 signaling pathway	12	12	3	0.008093	CASP3; CASP8; CCND1; CCNE1; CDK1; CDK2; CDK6; CHEK2; GADD45A; IGFBP3; PTEN; THBS1;
Cytokine Signaling in Immune system	56	56	6	0.012262	BLNK; CAMK2A; CAMK2D; CAMK2G; CASP1; CBL; CD44; CDK1; CHUK; CRK; CRKL; EIF2AK2; FYN;

					HRAS; IFNAR1; IL6ST; IL7R; IRF1; IRS1; JAK1; JAK2; LCK; LYN; MAP2K1; MAP2K2; MAP2K4; MAP3K3; MAP3K7; MAP3K8; MAPK1; NFKB1; NFKB2; PELI1; PELI2; PIK3CB; PIK3CD; PIK3R1; PIK3R2; PLCG1; PRKACA; PRKCD; PTK2B; PTPN1; RAF1; SHC1; SOCS3; SOS1; STAT1; STAT3; STAT5B; TAB1; TAB3; TOLLIP; TRAF6; YWHAB; YWHAZ;
E2F transcription factor network	5	5	2	0.012642	CCNE1; CDC25A; CDK1; CDK2; SIRT1;
Interferon alpha/beta signaling	5	5	2	0.012642	IFNAR1; IRF1; JAK1; PTPN1; SOCS3;
S Phase	5	5	2	0.012642	CCND1; CCNE1; CDC25A; CDK2; CDK7;
Signaling by Interleukins	44	44	5	0.018475	BLNK; CASP1; CBL; CDK1; CHUK; CRK; CRKL; FYN; HRAS; IL6ST; IL7R; JAK1; JAK2; LCK; LYN; MAP2K1; MAP2K2; MAP2K4; MAP3K3; MAP3K7; MAP3K8;

					MAPK1; NFKB1; NFKB2; PELI1; PELI2; PIK3CB; PIK3CD; PIK3R1; PIK3R2; PRKACA; PTK2B; RAF1; SHC1; SOCS3; SOS1; STAT3; STAT5B; TAB1; TAB3; TOLLIP; TRAF6; YWHAB; YWHAZ;
Degradation of the extracellular matrix	6	6	2	0.018538	CASP3; CD44; CTSB; CTSL1; CTSS; MMP8;
ERK activation	6	6	2	0.018538	CDK1; JAK1; JAK2; MAP2K1; MAP2K2; MAPK1;
TRAF6 mediated IRF7 activation	6	6	2	0.018538	EP300; IKBKE; IRF1; TBK1; TRAF2; TRAF6;
p73 transcription factor network	6	6	2	0.018538	ABL1; CDK2; CDK6; EP300; JAK1; SIRT1;
Cell cycle	16	16	3	0.018709	ABL1; CCND1; CCNE1; CDC25A; CDK1; CDK2; CDK6; CDK7; CHEK2; EP300; GADD45A; GSK3B; SMAD2; SMAD3; YWHAB; YWHAZ;
IFN-gamma pathway	17	17	3	0.022178	AKT1; CASP1; CBL; CEBPB; CRKL; DAPK1; IRF1; JAK1; JAK2; MAP2K1; MAP3K11; MAP3K1; MTOR; PIK3R1;

					PRKCD; STAT1; STAT3;
Caspase cascade in apoptosis	7	7	2	0.025372	BIRC3; CASP1; CASP3; CASP6; CASP8; PRF1; SREBF1;
Cell death signalling via NRAGE, NRIF and NADE	7	7	2	0.025372	CASP3; MAPK8; NGEF; NGFR; RAC1; SOS1; TRAF6;
Amyotrophic lateral sclerosis (ALS)	8	8	2	0.033073	CASP1; CASP3; CAT; MAP2K3; MAP3K5; MAPK14; RAB5A; RAC1;
FOXM1 transcription factor network	8	8	2	0.033073	CCND1; CDK1; CDK2; CHEK2; FOS; GSK3A; MAP2K1; NFATC3;
Retinoic acid receptors-mediated signaling	8	8	2	0.033073	AKT1; CDK1; CDK7; MAPK14; MAPK8; NCOA1; PRKACA; PRKCA;
MyD88 cascade initiated on plasma membrane	35	35	4	0.035765	APP; ATF2; CDK1; CHUK; CREB1; DUSP6; FOS; JAK1; JAK2; JUN; MAP2K1; MAP2K2; MAP2K3; MAP2K4; MAP3K1; MAP3K7; MAPK1; MAPK14; MAPK8; MAPKAPK2; MAPKAPK3; NFKB1; NFKB2; NFKBIA; PELI1; PELI2; PPP2CA; PPP2R5D; RPS6KA1; RPS6KA3;

					RPS6KA5; TAB1; TAB3; TLR5; TRAF6;
Toll Like Receptor 10 (TLR10) Cascade	35	35	4	0.035765	APP; ATF2; CDK1; CHUK; CREB1; DUSP6; FOS; JAK1; JAK2; JUN; MAP2K1; MAP2K2; MAP2K3; MAP2K4; MAP3K1; MAP3K7; MAPK1; MAPK14; MAPK8; MAPKAPK2; MAPKAPK3; NFKB1; NFKB2; NFKBIA; PELI1; PELI2; PPP2CA; PPP2R5D; RPS6KA1; RPS6KA3; RPS6KA5; TAB1; TAB3; TLR5; TRAF6;
Toll Like Receptor 5 (TLR5) Cascade	35	35	4	0.035765	APP; ATF2; CDK1; CHUK; CREB1; DUSP6; FOS; JAK1; JAK2; JUN; MAP2K1; MAP2K2; MAP2K3; MAP2K4; MAP3K1; MAP3K7; MAPK1; MAPK14; MAPK8; MAPKAPK2; MAPKAPK3; NFKB1; NFKB2; NFKBIA; PELI1; PELI2; PPP2CA; PPP2R5D; RPS6KA1; RPS6KA3; RPS6KA5; TAB1; TAB3; TLR5; TRAF6;

MyD88 dependent cascade initiated on endosome	36	36	4	0.039263	APP; ATF2; CDK1; CHUK; CREB1; DUSP6; FOS; JAK1; JAK2; JUN; MAP2K1; MAP2K2; MAP2K3; MAP2K4; MAP3K1; MAP3K7; MAPK1; MAPK14; MAPK8; MAPKAPK2; MAPKAPK3; NFKB1; NFKB2; NFKBIA; PELI1; PELI2; PPP2CA; PPP2R5D; RPS6KA1; RPS6KA3; RPS6KA5; TAB1; TAB3; TLR7; TLR8; TRAF6;
TRAF6 mediated induction of NFkB and MAP kinases upon TLR7/8 or 9 activation	36	36	4	0.039263	APP; ATF2; CDK1; CHUK; CREB1; DUSP6; FOS; JAK1; JAK2; JUN; MAP2K1; MAP2K2; MAP2K3; MAP2K4; MAP3K1; MAP3K7; MAPK1; MAPK14; MAPK8; MAPKAPK2; MAPKAPK3; NFKB1; NFKB2; NFKBIA; PELI1; PELI2; PPP2CA; PPP2R5D; RPS6KA1; RPS6KA3; RPS6KA5; TAB1; TAB3; TLR7; TLR8; TRAF6;

Toll Like Receptor 7/8 (TLR7/8) Cascade	36	36	4	0.039263	APP; ATF2; CDK1; CHUK; CREB1; DUSP6; FOS; JAK1; JAK2; JUN; MAP2K1; MAP2K2; MAP2K3; MAP2K4; MAP3K1; MAP3K7; MAPK1; MAPK14; MAPK8; MAPKAPK2; MAPKAPK3; NFKB1; NFKB2; NFKBIA; PELI1; PELI2; PPP2CA; PPP2R5D; RPS6KA1; RPS6KA3; RPS6KA5; TAB1; TAB3; TLR7; TLR8; TRAF6;
Cell cycle: g1/s check point	9	9	2	0.041573	ABL1; CCND1; CCNE1; CDC25A; CDK1; CDK2; CDK6; GSK3B; SMAD3;
RAF/MAP kinase cascade	9	9	2	0.041573	CDK1; HRAS; JAK1; JAK2; MAP2K1; MAP2K2; MAPK1; RAF1; YWHAB;
Androgen Receptor	37	37	4	0.042958	AKT1; ATF2; CASP1; CASP3; CASP8; CAV1; CCND1; CCNE1; CDC25A; CDC37; CDK1; CDK7; CDK9; CREB1; CTNNB1; EGFR; EP300; GAPDH; GSK3B; HSP90B1;

					HSPA4; HSPA5; HSPA8; HSPB1; IL6ST; JUN; NCOA1; PIK3R1; PIK3R2; PTEN; PTK2; PXN; SIRT1; SMAD3; SRC; STAT3; STUB1;
MyD88:Mal cascade initiated on plasma membrane	37	37	4	0.042958	APP; ATF2; BTK; CDK1; CHUK; CREB1; DUSP6; FOS; JAK1; JAK2; JUN; MAP2K1; MAP2K2; MAP2K3; MAP2K4; MAP3K1; MAP3K7; MAPK1; MAPK14; MAPK8; MAPKAPK2; MAPKAPK3; NFKB1; NFKB2; NFKBIA; PELI1; PELI2; PPP2CA; PPP2R5D; RPS6KA1; RPS6KA3; RPS6KA5; TAB1; TAB3; TIRAP; TLR6; TRAF6;
Toll Like Receptor 2 (TLR2) Cascade	37	37	4	0.042958	APP; ATF2; BTK; CDK1; CHUK; CREB1; DUSP6; FOS; JAK1; JAK2; JUN; MAP2K1; MAP2K2; MAP2K3; MAP2K4; MAP3K1; MAP3K7; MAPK1; MAPK14; MAPK8; MAPKAPK2; MAPKAPK3; NFKB1;

					NFKB2; NFKBIA; PELI1; PELI2; PPP2CA; PPP2R5D; RPS6KA1; RPS6KA3; RPS6KA5; TAB1; TAB3; TIRAP; TLR6; TRAF6;
Toll Like Receptor 9 (TLR9) Cascade	37	37	4	0.042958	APP; ATF2; CDK1; CHUK; CREB1; DUSP6; EEA1; FOS; JAK1; JAK2; JUN; MAP2K1; MAP2K2; MAP2K3; MAP2K4; MAP3K1; MAP3K7; MAPK1; MAPK14; MAPK8; MAPKAPK2; MAPKAPK3; NFKB1; NFKB2; NFKBIA; PELI1; PELI2; PPP2CA; PPP2R5D; RPS6KA1; RPS6KA3; RPS6KA5; TAB1; TAB3; TLR7; TLR8; TRAF6;
Toll Like Receptor TLR1:TLR2 Cascade	37	37	4	0.042958	APP; ATF2; BTK; CDK1; CHUK; CREB1; DUSP6; FOS; JAK1; JAK2; JUN; MAP2K1; MAP2K2; MAP2K3; MAP2K4; MAP3K1; MAP3K7; MAPK1; MAPK14; MAPK8; MAPKAPK2; MAPKAPK3; NFKB1;

					NFKB2; NFKBIA; PELI1; PELI2; PPP2CA; PPP2R5D; RPS6KA1; RPS6KA3; RPS6KA5; TAB1; TAB3; TIRAP; TLR6; TRAF6;
Toll Like Receptor TLR6:TLR2 Cascade	37	37	4	0.042958	APP; ATF2; BTK; CDK1; CHUK; CREB1; DUSP6; FOS; JAK1; JAK2; JUN; MAP2K1; MAP2K2; MAP2K3; MAP2K4; MAP3K1; MAP3K7; MAPK1; MAPK14; MAPK8; MAPKAPK2; MAPKAPK3; NFKB1; NFKB2; NFKBIA; PELI1; PELI2; PPP2CA; PPP2R5D; RPS6KA1; RPS6KA3; RPS6KA5; TAB1; TAB3; TIRAP; TLR6; TRAF6;
Cell Cycle	22	22	3	0.044538	CCND1; CCNE1; CDC25A; CDK1; CDK2; CDK6; CDK7; CSNK1D; CSNK1E; CSNK2A1; CSNK2A2; EP300; LPIN1; LPIN2; LPIN3; MAPK1; PPP1R12A; PPP2CA; PPP2R5D; PRKACA; PRKCA; TAOK1;

Cell Cycle, Mitotic	22	22	3	0.044538	CCND1; CCNE1; CDC25A; CDK1; CDK2; CDK6; CDK7; CSNK1D; CSNK1E; CSNK2A1; CSNK2A2; EP300; LPIN1; LPIN2; LPIN3; MAPK1; PPP1R12A; PPP2CA; PPP2R5D; PRKACA; PRKCA; TAOK1;

Table S3: Upregulated signaling pathways in pH1N1-infected alveolar epithelial cells at the alveolar-capillary barrier as determined by temporal kinome analysis. Pathways represent those that were found to be activated in pH1N1 infected cells as compared to mock-infected controls at 24 h post-MRSA addition. Thresholds for pathways over-representation analysis were fold-change ≥1.5, p-value ≤ 0.05.

Pathway Name	Pathway uploaded proteins	Genes in InnateDB for this entity	Pathway up-regulated genes count	Pathway up-regulated p-value	Gene Symbols
IFN-gamma pathway	17	17	4	4.66E-04	AKT1; CASP1; CBL; CEBPB; CRKL; DAPK1; IRF1; JAK1; JAK2; MAP2K1; MAP3K11; MAP3K1; MTOR; PIK3R1; PRKCD; STAT1; STAT3;
D4gdi signaling pathway	4	4	2	0.003405	CASP1; CASP3; JUN; RHOA;
Internal ribosome entry pathway	4	4	2	0.003405	CASP1; CASP3; CASP6; CASP8;
Cytokine Signaling in Immune system	56	56	5	0.007846	BLNK; CAMK2A; CAMK2D; CAMK2G; CASP1; CBL; CD44; CDK1; CHUK; CRK; CRKL; EIF2AK2; FYN; HRAS; IFNAR1; IL6ST; IL7R; IRF1; IRS1; JAK1; JAK2; LCK; LYN; MAP2K1; MAP2K2; MAP2K4; MAP3K3; MAP3K7; MAP3K8; MAPK1; NFKB1; NFKB2; PELI1; PELI2; PIK3CB; PIK3CD;

					PIK3R1; PIK3R2; PLCG1; PRKACA; PRKCD; PTK2B; PTPN1; RAF1; SHC1; SOCS3; SOS1; STAT1; STAT3; STAT5B; TAB1; TAB3; TOLLIP; TRAF6; YWHAB; YWHAZ;
Caspase cascade in apoptosis	7	7	2	0.011422	BIRC3; CASP1; CASP3; CASP6; CASP8; PRF1; SREBF1;
Amyotrophic lateral sclerosis (ALS)	8	8	2	0.015016	CASP1; CASP3; CAT; MAP2K3; MAP3K5; MAPK14; RAB5A; RAC1;
Signaling by FGFR1 mutants	9	9	2	0.019035	BCR; FGF20; FGFR1; FGFR1OP2; PIK3R1; PLCG1; STAT1; STAT3; STAT5B;
Oncostatin M	27	27	3	0.025447	AKT1; CASP3; CDK2; CEBPB; EP300; FOS; GRB2; HRAS; IL6ST; IRS1; JAK1; JAK2; MAPK1; MTOR; NFKB1; NFKBIA; PIK3R1; PTK2B; PXN; RAF1; SHC1; SOCS3; SOS1; SRC; STAT1; STAT3; STAT5B;
Factors involved in megakaryocyte	11	11	2	0.02827	ABL1; CDK2; DOCK6; EP300; IRF1; JAK2;

development and platelet production					KIF15; PRKACA; PRKAR1B; RAB5A; RAC1;
Pathways in cancer	77	77	5	0.030483	ABL1; AKT1; AKT3; BCR; BIRC3; BRAF; CASP3; CASP8; CBL; CCND1; CCNE1; CDK2; CDK6; CHUK; CKS2; CRK; CRKL; CSF1R; CTNNB1; DAPK1; EGFR; EP300; ETS1; FGF20; FGFR1; FGFR2; FGFR3; FLT3; FOS; FZD1; GRB2; GSK3B; HIF1A; HRAS; HSP90AB1; HSP90B1; ITGA6; JAK1; JUN; KIT; LEF1; MAP2K1; MAP2K2; MAPK1; MAPK8; MET; MTOR; NFKB1; NFKB2; NFKBIA; NOS2; PDGFRA; PDGFRB; PIK3CB; PIK3CD; PIK3CG; PIK3R1; PIK3R2; PLCG1; PLCG2; PPARG; PRKCA; PTEN; PTK2; RAC1; RAF1; RASSF5; RHOA; SMAD2; SMAD3; SOS1; STAT1; STAT3;

					STAT5B; TGFBR1; TRAF2; TRAF6;
Glucocorticoid receptor regulatory network	12	12	2	0.033448	AKT1; CREB1; FKBP5; FOS; GSK3B; IRF1; JUN; MAPK8; NCOA1; NFATC1; NFKB1; STAT1;
p53 signaling pathway	12	12	2	0.033448	CASP3; CASP8; CCND1; CCNE1; CDK1; CDK2; CDK6; CHEK2; GADD45A; IGFBP3; PTEN; THBS1;
Interferon Signaling	14	14	2	0.044833	CAMK2A; CAMK2D; CAMK2G; CD44; EIF2AK2; IFNAR1; IRF1; JAK1; JAK2; PLCG1; PRKCD; PTPN1; SOCS3; STAT1;

Table S4: Upregulated signaling pathways in MRSA-infected alveolar epithelial cells at the alveolar-capillary barrier. Pathways represent those that were found to be activated in MRSA infected cells as compared to mock-infected controls at 24 h post-MRSA addition. Thresholds for pathways over-representation analysis were fold-change ≥1.5, p-value ≤ 0.05.

Pathway Name	Pathway uploaded proteins	Genes in InnateDB for this entity	Pathway up-regulated genes count	Pathway up-regulated p-value	Gene Symbols
Regulation of Insulin-like Growth Factor (IGF) transport and uptake by Insulin-like Growth Factor Binding Proteins (IGFBPs)	4	4	2	1.59E-04	IGFBP1; IGFBP2; IGFBP3; IGFBP5;
Metabolism of proteins	20	20	2	0.004914	ACO2; ATP5B; CS; EIF2AK3; EIF4EBP1; GSK3A; HSP90B1; HSPA5; HSPA9; HSPD1; IGFBP1; IGFBP2; IGFBP3; IGFBP5; PDIA3; PGM3; PROS1; RPLP2; SHC1; THBS1;
IGF1 signaling pathway	22	22	2	0.005958	AKT1; AKT3; BRAF; GRB2; HRAS; IGFBP1; IGFBP2; IGFBP3; IGFBP5; IRS1; MAP2K1; MAP2K2; MAPK1; PDPK1; PIK3CB; PIK3CD; PIK3R1; PIK3R2; RAF1; SHC1; SHC3; SOS1;
Interleukin-1 processing	3	3	1	0.018828	CASP1; NFKB1; NFKB2;

PERK regulates gene expression	3	3	1	0.018828	EIF2AK3; HSPA5; IGFBP1;
D4gdi signaling pathway	4	4	1	0.025051	CASP1; CASP3; JUN; RHOA;
Internal ribosome entry pathway	4	4	1	0.025051	CASP1; CASP3; CASP6; CASP8;
Cellular roles of Anthrax toxin	5	5	1	0.031248	CASP1; MAP2K1; MAP2K2; MAP2K3; MAP2K4;
Inflammasomes	5	5	1	0.031248	APP; CASP1; HSP90AB1; NLRP3; PSTPIP1;
The NLRP3 inflammasome	5	5	1	0.031248	APP; CASP1; HSP90AB1; NLRP3; PSTPIP1;
Cytosolic DNA-sensing pathway	6	6	1	0.037418	CASP1; CHUK; IKBKE; NFKB1; NFKBIA; TBK1;
Unfolded Protein Response (UPR)	6	6	1	0.037418	EIF2AK3; GSK3A; HSP90B1; HSPA5; IGFBP1; SHC1;
Caspase cascade in apoptosis	7	7	1	0.043562	BIRC3; CASP1; CASP3; CASP6; CASP8; PRF1; SREBF1;
FOXA2 and FOXA3 transcription factor networks	7	7	1	0.043562	ACADM; AKT1; ALDOB; CREB1; HADH; IGFBP1; PKLR;
Amyotrophic lateral sclerosis (ALS)	8	8	1	0.04968	CASP1; CASP3; CAT; MAP2K3; MAP3K5; MAPK14; RAB5A; RAC1;

Table S5: Upregulated signaling pathways in pH1N1-MRSA-infected pulmonary endothelial cells at the alveolar-capillary barrier. Pathways represent those that were found to be activated in pH1N1-MRSA co-infected endothelial cells as compared to mock-infected controls at 24 h post-MRSA addition. Thresholds for pathway over-representation analysis were fold-change ≥1.5, p-value ≤0.05.

Pathway Name	Pathway uploaded proteins	Genes in InnateDB for this entity	Pathway up-regulated genes count	Pathway up-regulated p-value	Gene Symbols
Hedgehog 'off' state	4	4	3	7.05E-04	CSNK1A1; GSK3B; PRKACA; PRKAR1B;
Signaling by Hedgehog	4	4	3	7.05E-04	CSNK1A1; GSK3B; PRKACA; PRKAR1B;
Hedgehog signaling pathway	6	6	3	0.003249	CSNK1A1; CSNK1D; CSNK1E; CSNK1G1; GSK3B; PRKACA;
Class I MHC mediated antigen processing & presentation	8	8	3	0.008394	CTSL1; CTSS; HSPA5; NCF1; PDIA3; SOCS3; STUB1; TRIM36;
Degradation of GLI2 by the proteasome	3	3	2	0.009664	CSNK1A1; GSK3B; PRKACA;
GLI3 is processed to GLI3R by the proteasome	3	3	2	0.009664	CSNK1A1; GSK3B; PRKACA;
Transcription factor creb and its extracellular signals	17	17	4	0.013452	AKT1; CAMK2A; CAMK2D; CAMK2G; CREB1; GRB2; HRAS; MAPK1; MAPK14; PDPK1; PIK3R1; PRKAR1B; PRKCA; RPS6KA1; RPS6KA5; SHC1; SOS1;
Cellular responses to stress	36	36	6	0.013505	CAMK2A; CAMK2D;

					CAMK2G; CAT; CCNE1; CDK2; CDK6; CEBPB; EP300; ETS1; FOS; GSK3B; HIF1A; HSP90AB1; HSPA8; IGFBP7; JUN; MAP2K3; MAP2K4; MAP3K5; MAPK1; MAPK14; MAPK8; MAPKAPK2; MAPKAPK3; MAPKAPK5; MLST8; MTOR; NFKB1; PRDX2; RPS6KA1; RPS6KA3; RPTOR; SIRT1; STAT3; TNIK;
VEGFR3 signaling in lymphatic endothelium	10	10	3	0.016598	AKT1; CREB1; CRK; FLT4; GRB2; MAP2K4; PIK3R1; RPS6KA1; SHC1; SOS1;
Antigen processing-cross presentation	4	4	2	0.018624	CTSL1; CTSS; NCF1; PDIA3;
Beta-catenin phosphorylation cascade	4	4	2	0.018624	CSNK1A1; GSK3B; PPP2CA; PPP2R5D;
Circadian Clock	11	11	3	0.021926	ATF2; CPT1A; CREB1; CSNK1D; CSNK1E; EP300; HIF1A; NCOA1; PPARA;

					PPARGC1A; SREBF1;
Factors involved in megakaryocyte development and platelet production	11	11	3	0.021926	ABL1; CDK2; DOCK6; EP300; IRF1; JAK2; KIF15; PRKACA; PRKAR1B; RAB5A; RAC1;
CREB phosphorylation	5	5	2	0.029912	CREB1; MAPKAPK2; RPS6KA1; RPS6KA3; RPS6KA5;
Tnfr1 signaling pathway	5	5	2	0.029912	BIRC3; CASP3; CASP8; MAP2K4; TRAF2;
Role of Calcineurin-dependent NFAT signaling in lymphocytes	13	13	3	0.035082	CASP3; CSNK1A1; CSNK2A1; GSK3B; MAP3K1; MAP3K8; MAPK14; MAPK8; NFATC1; NFATC2; NFATC3; PIM1; PRKACA;
Signaling by Wnt	23	23	4	0.039044	AKT1; AP2M1; ARRB2; CAMK2A; CAV1; CSNK1A1; CSNK1E; CSNK2A1; CSNK2A2; CTNNB1; EP300; FZD1; GSK3B; LEF1; MAP3K7; NFATC1; PLCB1; PPP2CA; PPP2R5D; PRKCA; RAC1; RHOA; YWHAZ;

MAPK targets/ Nuclear events mediated by MAP kinases	14	14	3	0.042903	ATF2; CREB1; DUSP6; FOS; JUN; MAPK1; MAPK14; MAPK8; MAPKAPK2; PPP2CA; PPP2R5D; RPS6KA1; RPS6KA3; RPS6KA5;
P38 mapk signaling pathway	14	14	3	0.042903	ATF2; CREB1; HRAS; HSPB1; MAP2K4; MAP3K1; MAP3K5; MAPK14; MAPKAPK2; MAPKAPK5; MKNK1; RAC1; RPS6KA5; STAT1;
RNF mutants show enhanced WNT signaling and proliferation	14	14	3	0.042903	AKT1; CAV1; CSNK1A1; CSNK1E; CSNK2A1; CSNK2A2; CTNNB1; EP300; FZD1; GSK3B; LEF1; PPP2CA; PPP2R5D; YWHAZ;
Signaling by WNT in cancer	14	14	3	0.042903	AKT1; CAV1; CSNK1A1; CSNK1E; CSNK2A1; CSNK2A2; CTNNB1; EP300; FZD1; GSK3B; LEF1; PPP2CA; PPP2R5D; YWHAZ;

TCF dependent signaling in response to WNT	14	14	3	0.042903	AKT1; CAV1; CSNK1A1; CSNK1E; CSNK2A1; CSNK2A2; CTNNB1; EP300; FZD1; GSK3B; LEF1; PPP2CA; PPP2R5D; YWHAZ;
XAV939 inhibits tankyrase, stabilizing AXIN	14	14	3	0.042903	AKT1; CAV1; CSNK1A1; CSNK1E; CSNK2A1; CSNK2A2; CTNNB1; EP300; FZD1; GSK3B; LEF1; PPP2CA; PPP2R5D; YWHAZ;
misspliced LRP5 mutants have enhanced beta-catenin-dependent signaling	14	14	3	0.042903	AKT1; CAV1; CSNK1A1; CSNK1E; CSNK2A1; CSNK2A2; CTNNB1; EP300; FZD1; GSK3B; LEF1; PPP2CA; PPP2R5D; YWHAZ;
AMER1 mutants destabilize the destruction complex	6	6	2	0.043243	CSNK1A1; CTNNB1; GSK3B; LEF1; PPP2CA; PPP2R5D;
APC truncation mutants are not K63 polyubiquitinated	6	6	2	0.043243	CSNK1A1; CTNNB1; GSK3B; LEF1; PPP2CA; PPP2R5D;
APC truncation mutants have impaired AXIN binding	6	6	2	0.043243	CSNK1A1; CTNNB1; GSK3B;

					LEF1; PPP2CA; PPP2R5D;
AXIN missense mutants destabilize the destruction complex	6	6	2	0.043243	CSNK1A1; CTNNB1; GSK3B; LEF1; PPP2CA; PPP2R5D;
AXIN mutants destabilize the destruction complex, activating WNT signaling	6	6	2	0.043243	CSNK1A1; CTNNB1; GSK3B; LEF1; PPP2CA; PPP2R5D;
Degradation of beta-catenin by the destruction complex	6	6	2	0.043243	CSNK1A1; CTNNB1; GSK3B; LEF1; PPP2CA; PPP2R5D;
Degradation of the extracellular matrix	6	6	2	0.043243	CASP3; CD44; CTSB; CTSL1; CTSS; MMP8;
S33 mutants of beta-catenin aren't phosphorylated	6	6	2	0.043243	CSNK1A1; CTNNB1; GSK3B; LEF1; PPP2CA; PPP2R5D;
S37 mutants of beta-catenin aren't phosphorylated	6	6	2	0.043243	CSNK1A1; CTNNB1; GSK3B; LEF1; PPP2CA; PPP2R5D;
S45 mutants of beta-catenin aren't phosphorylated	6	6	2	0.043243	CSNK1A1; CTNNB1; GSK3B; LEF1; PPP2CA; PPP2R5D;
Stathmin and breast cancer resistance to antimicrotubule agents	6	6	2	0.043243	CAMK2A; CAMK2D; CAMK2G; CDK1; PRKAR1B; STMN1;

T41 mutants of beta-catenin aren't phosphorylated	6	6	2	0.043243	CSNK1A1; CTNNB1; GSK3B; LEF1; PPP2CA; PPP2R5D;
TCF7L2 mutants don't bind CTBP	6	6	2	0.043243	CSNK1A1; CTNNB1; GSK3B; LEF1; PPP2CA; PPP2R5D;
deletions in the AMER1 gene destabilize the destruction complex	6	6	2	0.043243	CSNK1A1; CTNNB1; GSK3B; LEF1; PPP2CA; PPP2R5D;
deletions in the AXIN genes in hepatocellular carcinoma result in elevated WNT signaling	6	6	2	0.043243	CSNK1A1; CTNNB1; GSK3B; LEF1; PPP2CA; PPP2R5D;
misspliced GSK3beta mutants stabilize beta-catenin	6	6	2	0.043243	CSNK1A1; CTNNB1; GSK3B; LEF1; PPP2CA; PPP2R5D;
phosphorylation site mutants of CTNNB1 are not targeted to the proteasome by the destruction complex	6	6	2	0.043243	CSNK1A1; CTNNB1; GSK3B; LEF1; PPP2CA; PPP2R5D;
truncated APC mutants destabilize the destruction complex	6	6	2	0.043243	CSNK1A1; CTNNB1; GSK3B; LEF1; PPP2CA; PPP2R5D;
truncations of AMER1 destabilize the destruction complex	6	6	2	0.043243	CSNK1A1; CTNNB1; GSK3B; LEF1; PPP2CA; PPP2R5D;

Table S6: Upregulated signaling pathways in pH1N1-infected pulmonary endothelial cells at the alveolar-capillary barrier. Pathways represent those that were found to be activated in pH1N1 infected endothelial cells as compared to mock-infected controls at 24 h post-MRSA addition. Thresholds for pathway over-representation analysis were fold-change ≥1.5, p-value ≤0.05.

Pathway Name	Pathway uploaded proteins	Genes in InnateDB for this entity	Pathway up-regulated genes count	Pathway up-regulated p-value	Gene Symbols
Pyruvate metabolism	4	4	2	0.007759	LDHA; LDHB; PDK1; PDK4;

Table S7: Upregulated signaling pathways in MRSA-infected pulmonary endothelial cells at the alveolar-capillary barrier. Pathways represent those that were found to be activated in MRSA infected endothelial cells as compared to mock-infected controls at 24 h post-MRSA addition. Thresholds for pathway over-representation analysis were fold-change ≥1.5, p-value ≤0.05.

Pathway Name	Pathway uploaded proteins	Genes in InnateDB for this entity	Pathway up-regulated genes count	Pathway up-regulated p-value	Gene Symbols
Hedgehog signaling pathway	6	6	4	1.78E-04	CSNK1A1; CSNK1D; CSNK1E; CSNK1G1; GSK3B; PRKACA;
Hedgehog 'off' state	4	4	3	8.70E-04	CSNK1A1; GSK3B; PRKACA; PRKAR1B;
Signaling by Hedgehog	4	4	3	8.70E-04	CSNK1A1; GSK3B; PRKACA; PRKAR1B;
Degradation of GLI2 by the proteasome	3	3	2	0.011089	CSNK1A1; GSK3B; PRKACA;
GLI3 is processed to GLI3R by the proteasome	3	3	2	0.011089	CSNK1A1; GSK3B; PRKACA;
Metabolism of lipids and lipoproteins	3	3	2	0.011089	Dgat1; Fdx1; Ptgs2;
PKA-mediated phosphorylation of CREB	3	3	2	0.011089	CREB1; PRKACA; PRKAR1B;
Transcription regulation by methyltransferase of carm1	3	3	2	0.011089	CREB1; EP300; PRKAR1B;
Beta-catenin phosphorylation cascade	4	4	2	0.021308	CSNK1A1; GSK3B; PPP2CA; PPP2R5D;
CREB phosphorylation through the activation of CaMKII	4	4	2	0.021308	CAMK2A; CAMK2D; CAMK2G; CREB1;
CRMPs in Sema3A signaling	4	4	2	0.021308	DPYSL2; DPYSL3; FYN; GSK3B;
GPCR Dopamine D1like receptor signaling pathway	4	4	2	0.021308	CREB1; PPP2R5D; PRKACA; PRKAR1B;

Metabolism	4	4	2	0.021308	Dgat1; Fdx1; Pfkfb1; Ptgs2;
Presenilin action in Notch and Wnt signaling	12	12	3	0.033806	CCND1; CSNK1A1; CSNK2A1; CTNNB1; FOS; FZD1; GSK3B; JUN; MAP3K7; MAPK1; PPP2R5D; TAB1;
Repression of pain sensation by the transcriptional regulator dream	5	5	2	0.034124	CREB1; PRKACA; PRKAR1B; PRKCA; PRKCD;
Vasopressin-regulated water reabsorption	5	5	2	0.034124	CREB1; PRKACA; PRKAR1B; PRKCA; PRKCD;
AMER1 mutants destabilize the destruction complex	6	6	2	0.049193	CREB1; FOS; JUN; PRKAR1B; RPS6KA3;
APC truncation mutants are not K63 polyubiquitinated	6	6	2	0.049193	CREB1; DYNC1LI1; PRKACA; RAB11A; RAB5A;
APC truncation mutants have impaired AXIN binding	6	6	3	0.049193	AKT1; CAMK2A; CCND1; CREB1; GRB2; GSK3B; PDPK1; PIK3R1; PLCG1; PRKCD; SHC1; SOS1; SRC;
AXIN missense mutants destabilize the destruction complex	6	6	4	0.049193	AKT1; AP2M1; ARRB2; CAMK2A; CAV1; CSNK1A1; CSNK1E; CSNK2A1; CSNK2A2; CTNNB1; EP300; FZD1; GSK3B; LEF1; MAP3K7; NFATC1;

					PLCB1; PPP2CA; PPP2R5D; PRKCA; RAC1; RHOA; YWHAZ;
AXIN mutants destabilize the destruction complex, activating WNT signaling	6	6	2	0.049193	CSNK1A1; CTNNB1; GSK3B; LEF1; PPP2CA; PPP2R5D;
Ca-calmodulin-dependent protein kinase activation	6	6	2	0.049193	CSNK1A1; CTNNB1; GSK3B; LEF1; PPP2CA; PPP2R5D;
Degradation of beta-catenin by the destruction complex	6	6	2	0.049193	CSNK1A1; CTNNB1; GSK3B; LEF1; PPP2CA; PPP2R5D;
S33 mutants of beta-catenin aren't phosphorylated	6	6	2	0.049193	CSNK1A1; CTNNB1; GSK3B; LEF1; PPP2CA; PPP2R5D;
S37 mutants of beta-catenin aren't phosphorylated	6	6	2	0.049193	CSNK1A1; CTNNB1; GSK3B; LEF1; PPP2CA; PPP2R5D;
S45 mutants of beta-catenin aren't phosphorylated	6	6	2	0.049193	CAMK2A; CAMK2D; CAMK2G; CAMKK1; CAMKK2; CREB1;
Stathmin and breast cancer resistance to antimicrotubule agents	6	6	2	0.049193	CSNK1A1; CTNNB1; GSK3B; LEF1; PPP2CA; PPP2R5D;

T41 mutants of beta-catenin aren't phosphorylated	6	6	2	0.049193	CSNK1A1; CTNNB1; GSK3B; LEF1; PPP2CA; PPP2R5D;
TCF7L2 mutants don't bind CTBP	6	6	2	0.049193	CSNK1A1; CTNNB1; GSK3B; LEF1; PPP2CA; PPP2R5D;
Deletions in the AMER1 gene destabilize the destruction complex	6	6	2	0.049193	CSNK1A1; CTNNB1; GSK3B; LEF1; PPP2CA; PPP2R5D;
Deletions in the AXIN genes in hepatocellular carcinoma result in elevated WNT signaling	6	6	2	0.049193	CAMK2A; CAMK2D; CAMK2G; CDK1; PRKAR1B; STMN1;
Misspliced GSK3beta mutants stabilize beta-catenin	6	6	2	0.049193	CSNK1A1; CTNNB1; GSK3B; LEF1; PPP2CA; PPP2R5D;
Phosphorylation site mutants of CTNNB1 are not targeted to the proteasome by the destruction complex	6	6	2	0.049193	CSNK1A1; CTNNB1; GSK3B; LEF1; PPP2CA; PPP2R5D;
Truncated APC mutants destabilize the destruction complex	6	6	2	0.049193	CSNK1A1; CTNNB1; GSK3B; LEF1; PPP2CA; PPP2R5D;
Truncations of AMER1 destabilize the destruction complex	6	6	2	0.049193	CSNK1A1; CTNNB1; GSK3B; LEF1; PPP2CA; PPP2R5D;
